# The significance of immunonutrition nutritional support in patients undergoing postoperative adjuvant chemotherapy for lung cancer: case–control study

**DOI:** 10.1186/s12957-023-03073-y

**Published:** 2023-06-19

**Authors:** Tevfik Ilker Akcam, Ahmet Kayahan Tekneci, Onder Kavurmaci, Ali Ozdil, Ayse Gul Ergonul, Kutsal Turhan, Alpaslan Cakan, Ufuk Cagirici

**Affiliations:** 1 Department of Thoracic Surgery, Ege University School of Medicine, İzmir, Turkey; 2 Department of Thoracic Surgery, Health Sciences University İzmir Tepecik Education and Research Hospital, İzmir, Turkey; 3 Department of Thoracic Surgery, SBU Bozyaka İzmir Training and Research Hospital, İzmir, Turkey

**Keywords:** Lung cancer, Nutrition, Resection, Adjuvant chemotherapy

## Abstract

**Background:**

In this study, the effect of postoperative early nutritional supplementation on the course of the disease was investigated in patients who were operated for non-small cell lung cancer and received adjuvant chemotherapy.

**Methods:**

The study examined the data of patients who anatomical pulmonary resection for non-small cell lung cancer and who were treated with adjuvant chemotherapy at our clinic between January 2014 and January 2020. Patients who received early postoperative nutritional supplements and those who continued with a normal diet were compared in terms of complications, mortality, recurrence, and survival.

**Results:**

The study sample consisted of 68 (84%) male and 13 (16%) female patients, and the mean duration of postoperative follow-up was 31.6 ± 17.9 (4–75) months. Metastasis was identified in eight (17.4%) patients in Grup_NS (Nutritional Supplements)_ compared to 10 (28.6%) patients in Group_C (Control)_ (*p* = 0.231). Of the total, 11 (23.9%) patients died in Group_NS_ compared to 13 (37.1%) in Group_C_ (*p* = 0.196). Mean survival was 58.9 ± 3.8 (95% *CI*: 4.0–75.0) months in Group_NS_ compared to 43.5 ± 4.6 (95% *CI*: 6.0–66.0) months in Group_C_ (*p* = 0.045).

**Conclusion:**

Early nutritional supplements should be considered as having a positive effect especially on survival in this specific patient group involving factors with high catabolic effects, such as neoplasia, operation, and chemotherapy together.

## Introduction

Lung cancer is currently the primary cause of cancer-related death [1, 2]. It is mainly classified into small cell lung cancer (SCLC) and non-small cell lung cancer (NSCLC), with non-small cell lung cancer (NSCLC) accounting for approximately 85% of all lung cancers [3]. Lung cancer treatments are based mainly on patient-specific characteristics, such as tumor histology, disease stage, age, pulmonary functions, and comorbidities [4]. Surgery is the most effective treatment method for early NSCLC patients, although patients identified with advanced-stage disease in postoperative staging may experience recurrence or distant metastasis, despite the operation. This suggests that the majority of patients have micrometastatic disease during resection, and those who are eligible are administered postoperative adjuvant chemotherapy, although the survival benefit of this approach is currently unknown [3, 5–7].

The increased catabolic process due to the operation and that is inherent in cancer patients increases the need for energy and other nutrients, such as protein. Malnutrition at this disease stage leads to a weakened immune system and causes such conditions as loss of weight and muscle mass. As a result, all stages of recovery are prolonged and become more complicated. Changes to dietary patterns by patients often fail to circumvent this outcome, indicating a need for nutritional supplements [8–10]. Several studies have found such supplements to improve survival and reduce potential complications during chemotherapy [10].

The present study compares the complications, mortality, recurrence, and survival outcomes of patients treated with postoperative nutritional supplements with those who received no such treatment.

## Materials and methods

The study included 202 patients being treated with postoperative adjuvant chemotherapy (CTx) among the 603 cases undergoing anatomic pulmonary resection due to primary lung cancer at our clinic between January 2014 and January 2020. Detailed inclusion criteria were established to homogenize the research population. Patients with a history of malnutrition and those diagnosed with diabetes were excluded from the study, and those who had received preoperative neoadjuvant chemotherapy were also excluded. Patients undergoing rethoracotomy or bronchoplastic procedures due to recurrence during follow-up were excluded. Patients with Stage I and Stage IV, as ascertained during surgical staging, were also excluded to ensure a homogeneous patient group. To avoid the effect of histological differences on the results, only patients diagnosed with lung adenocarcinoma or squamous cell carcinoma were included in the study. In total, 81 patients met the inclusion criteria and provided data on such parameters as age, sex, comorbidities, surgical procedures performed, postoperative complications (pneumonia, wound infection, prolonged air leakage, hemorrhage, electrolyte imbalance, impaired kidney function, neutropenia, etc.), length of hospital stay, histopathological examination results, tumor stage, survival, and postoperative recurrence and metastasis. Of the total, 46 of the patients who had received early postoperative nutritional supplements (nutrition program) with an immune modulating formula (enriched with arginine, omega-3 fatty acids, and nucleotides) were assigned to Grup_NS (Nutritional Supplements)_. The dose and duration of the treatment in patients receiving nutritional support were determined by a dietician. All of the patients who received nutritional support used the same supplemental nutritional content 3 times a day: in the morning, noon, and evening. A box of this nutritional supplements formula content was as follows: “Arginine, nucleotides, omega-3 fatty acids, protein, carbohydrates, fat, fiber, sodium, zinc, calcium, magnesium, iron, calories 341 kcal, etc.” This treatment regimen, which provides approximately 1023 kcal of additional nutritional support, was continued in all patients until the completion of adjuvant chemotherapy treatment. GrupNS consisted of patients who fully complied with the nutritional supplement regimen. In turn, 35 who were maintained on a normal diet without any additional nutritional products were named Grup_C_ (_Control_). Complications, mortality, recurrence, and survival in Group_NS_ and Group_C_ were analyzed retrospectively.

Written informed consent was obtained from each patient, and the study was conducted in accordance with the principles of the Declaration of Helsinki. The retrospective study was approved by the local ethics committee (no.: 20-9 T/35).

### Statistical analysis

Data were analyzed using the SPSS 25.0 (IBM statistics for Windows version 25, IBM Corporation, Armonk, New York, USA) and PAST (Hammer, Ø., Harper, D.A.T., Ryan, P.D. 2001. Paleontological Statistics) software packages. Quantitative data were expressed as mean ± standard deviation (SD) and as median range (minimum–maximum). Categorical data, in turn, were expressed as numbers (*n*) and percentages (%). Chi-square test, Fisher exact test, Student *T*-test, and Mann–Whitney *U*-test were used to evaluate the statistical differences between the groups. Survival was analyzed using the Kaplan–Meier method and a log-rank (Mantel-Cox) test. All data were evaluated at a 95% confidence interval, and a statistical significance level of *p* < 0.05 was determined.

## Results

The study sample of 81 patients comprised 68 (84%) males and 13 (16%) females, of which 32 (39.5%) were under the age of 60 years and 49 (60.5%) were aged 60 years and older; the mean age of all patients was 61.81 ± 9.2 (38–81) years. When the groups were evaluated according to gender distribution, while 42 (91.3%) of the cases were male in GroupNS, 26 (74.3%) were male in Group C. The male sex ratio was statistically higher in Group_NS_ (*p* = 0.039) (Table 3).

There were comorbidities in 54 (66.7%) patients; 32 (39.5%) had cardiac disease; 17 (21%) had endocrine disorders; 16 (19.7%) had a history of previous malignancies; and 13 (16%) were being followed up due to COPD (chronic obstructive pulmonary disease). A total of 19 (23.5%) patients had one or more comorbidities (urological, neurological, etc.) (Table 1).

**Table 1:** Demographic characteristics

**Variables**	**No. of patients (*n* = 81)**	**Frequency (%)**
**Sex**		
Male	68	84
Female	13	16
**Age**		
< 60 years (under 60 years of age)	32	39.5
≥ 60 years (aged 60 years and older)	49	60.5
Mean age of patients (mean ± SD, range) (years)	61.81 ± 9.2 (38–81)	
**Comorbidities**		
Cardiac	32	39.5
Endocrinological	17	20.9
Previous malignancy	16	19.7
COPD	13	16
Other	19	23.4
**Complications**		
Prolonged air leak	24	29.6
Cardiac	8	9.8
Blood product replacement	7	8.6
Pneumonia	5	6.1
Other	15	18.5
**Surgical procedures**		
RUL	19	23.5
RML	1	1.2
RLL	14	17.3
RUBL	3	3.7
RLBL	4	4.9
LUL	6	7.4
LLL	13	16
RP	5	6.2
LP	15	18.5
**Histopathological diagnosis**		
Adenocarcinoma	41	50.6
Squamous cell carcinoma	40	49.4
**Histopathological stage**		
Stage IIA	6	7.4
Stage IIB	41	50.6
Stage IIIA	28	34.6
Stage IIIB	6	7.4
**Metastasis**		
	18	22.2
**Mortality**		
	24	29.6
**Nutritional status**		
Nutritional supplement	46	56.8
Normal diet	35	43.2
**Mean length of hospital stay (LOS) (days)**	8.26 ± 0.56 (3–27 ± 5.03)/days	

*Abbreviations*: *COPD* Chronic obstructive pulmonary disease, *LLL* Left lower lobectomy, *LP* Left pneumonectomy, *LUL* Left upper lobectomy, *RLBL* Right lower bilobectomy, *RLL* Right lower lobectomy, *RML* Right middle lobectomy, *RP* Right pneumonectomy, *RUBL* Right upper bilobectomy, *RUL* Right upper lobectomy

All patients underwent anatomic resection, while 61 (75.3%) underwent a lobectomy and 20 (24.7%) a pneumonectomy. The most common type of anatomic resection was a right upper lobectomy, which was performed in 19 patients (23.5%) (Table 1). A pneumonectomy was performed in 17 (37%) patients in Group_NS_, compared to 3 (8.6%) patients in Group_C_. The rate of pneumonectomy was statistically significantly higher in Group_NS_ (*p* = 0.003) (Table 3).

The results of a histopathological examination revealed adenocarcinoma in 41 (50.6%) patients and squamous cell carcinoma in 40 (49.4%) patients. Adenocarcinoma was identified in 18 (39.1%) and 23 (65.7%) patients in Group_NS_ and Group_C_, respectively. The rate of squamous cell carcinoma was statistically significantly higher in GroupNS (*p* = 0.018) (Table 3).

Based on the 8th edition of the disease staging system, six (7.4%) patients were identified with Stage IIA, 41 (50.6%) with Stage IIB, 28 (34.6%) with Stage IIIA, and six (7.4%) with Stage IIIB (Table 1). Group_NS_ contained 25 (54.3%) patients with Stage II, two (4.4%) with Stage IIA, 23 (50%) with Stage IIB, 21 (45.7%) with Stage III, 15 (32.6%) with Stage IIIA, and six (13%) with Stage IIIB. In Group_C_, in turn, 22 (61.1%) patients were at Stage II, four (11.4%) were at Stage IIA, 18 (51.5%) were at Stage IIB, and 13 (37.1%) were at Stage III (all of Stage IIIA). There was no statistically significant difference in the staging results of the two groups of patients (*p* = 0.110) (Table 2).

**Table 2:** Demographic data of groups according to nutritional status and statistical differences between groups

**Variables**	**Nutritional supplement**		**Normal diet**		* p*-value
	No. of patients (*n* = 46)	Frequency (%)	No. of patients (*n* = 35)	Frequency (%)	
**Sex**					
Male	42	91.3	26	74.3	**0.039**
Female	4	8.7	9	25.7	
**Age**					
Mean age of patients (mean ± SD, range) (years) 62 ± 8.2 (48–81)			61.57 ± 10.5 (38–77)		0.689
**Comorbidities**					
	32	69.6	22	62.9	0.526
**Histopathological diagnosis**					
Adenocarcinoma	18	39.1	23	65.7	**0.018**
Squamous cell carcinoma	28	60.9	12	34.3	
**Histopathological stage**					
Stage IIA	2	4.3	4	11.4	0.110
Stage IIB	23	50	8	51.4	
Stage IIIA	15	32.6	13	37.1	
Stage IIIB	6	13	-	-	
**Surgical procedures**					
Lobar resection	29	63	32	91.4	
Pneumonectomy	17	37	3	8.6	**0.003**
**Complications**					
	23	50	16	45.7	0.702
**Metastasis**					
	8	17.4	10	28.6	0.231
**Mortality**					
	11	23.9	13	37.1	0.196
**Mean length of hospital stay (LOS) (days)**	8.15 ± 0.73 (3–27 ± 5)		8.4 ± 0.87 (3–26 ± 5.14)		0.973

The length of hospital stay was 8.3 ± 0.6 (3–27) days for all patients, with 8.15 ± 0.7 days (3–27) in Group_NS_ compared to 8.4 ± 0.9 days (3–23) in Group_C_. There was no statistically significant difference in age distribution, comorbidity rate, and length of hospital stay between Group_NS_ and Group_C_ (*p* = 0.689, *p* = 0.526, *p* = 0.973) (Table 2).

The adjuvant chemotherapy protocols were initiated at the end of first postoperative month. The mean duration of postoperative follow-up was 31.6 ± 17.9 (4–75) months.

Of the total, 39 (48.1%) patients developed early postoperative complications, with complications identified in 23 (50%) patients in Group_NS_ versus 16 (45.7%) patients in Group_C_. There was, however, no statistically significant difference in the incidence of early postoperative complications between the two groups (*p* = 0.702). The most common complication was prolonged air leakage in both groups, being identified in 11 (23.9%) patients in Group_NS_ and 13 (37.1%) patients in Group_C_, resulting in a total of 24 (29.5%) patients (Table 1). Of the total, five (14.2%) patients developed cardiac complications in Group_C_, compared to three (6.5%) patients in Group_NS_.

Among the total cases, 18 (22.2%) were radiologically identified to have metastasis during follow-up, while no metastasis was identified in 63 (77.8%) patients, with eight (17.4%) patients developing metastasis in Group_NS_ compared to 10 (28.6%) patients in Group_C_. Although metastasis was identified more in Group_C_, there was no statistically significant difference between the two groups in this regard (*p* = 0.231).

Of the total sample, 24 (29.6%) patients died, and 57 (70.4%) survived. Of those that died, 11 (23.9%) were in in Group_NS_, and 13 (37.1%) were in in Group_C_. There was no statistically significant difference in mortality between the two groups (*p* = 0.196) (Table 3).

**Table 3:** Statistical table of patient groups according to variables

**Relevant factors**	**Nutritional status** ***n* (%)**		***p*-value**
	**Group**_**NS**_	**Group**_**Control**_	
**Pneumonectomy (**surgical procedures)	17 (37%)	3 (8.6%)	**0.003**
**Complications**	23 (50%)	16 (45.7%)	0.702
**Metastasis**	8 (17.4%)	10 (28.6%)	0.231
**Mortality**	11 (23.9%)	13 (37.1%)	0.196
**Mean length of hospital stay (LOS) (days)**	8.15 ± 0.73 (3–27)	8.4 ± 0.87 (3–26)	0.973

*Abbreviation*: *NS* nutritional supplements

The effects of male gender, pneumonetomy procedure, and the presence of squamous cell carcinoma parameters on survival, which were found to be statistically significantly different between the two groups, were analyzed. The mean survival time in males was 50.86 ± 3.74 (95% *CI*: 4.0–75.0) months, while it was 59.10 ± 4.36 (95% *CI*: 18.0–66.0) months in females. The difference between the two groups was not statistically significant (*p* = 0.110) (Table 4). The mean survival time in patients who underwent the pneumonectomy procedure was 50.69 ± 3.94 (95% *CI*: 4.0–75.0) months, while it was 61.99 ± 5.45 (95% *CI*: 5.0–75.0) months in patients who underwent the non-pneumonectomy. The difference between the two groups was not statistically significant (*p* = 0.151) (Table 4). The mean survival time in patients with squamous cell carcinoma was 56.02 ± 4.22 (95% *CI*: 4.0–74.0) months, while it was 51.24 ± 4.96 (95% *CI*: 6.0–75.0) months in adenocarcinoma. The difference between the two groups was not statistically significant (*p* = 0.308) (Table 4). As a result, it was determined that although male gender, squamous cell carcinoma, and pneumonectomy procedures were more common in Group_NS_, these parameters had no effect on survival.

**Table 4:** Results of survival analysis

**Relevant factors**	***n* (%)**	**Mean survival (mean ± SD, range) (months)**	***p*-value**
**Nutritional status**			
Group_NS_	46 (56.8%)	58.91 ± 3.83 (4–75 ± 18.79)	**0.045**
Group_Control_	35 (43.2%)	43.49 ± 4.61 (6–66 ± 15.7)	-
**Sex**			
Male	68 (84%)	50.86 ± 3.74 (4–75 ± 17.97)	0.110
Female	13 (16%)	59.10 ± 4.36 (18–66 ± 15.84)	
**Histopathological diagnosis**			
Adenocarcinoma	41 (50.6%)	56.02 ± 4.22 (4–74 ± 17.72)	0.308
Squamous cell carcinoma	40 (49.4%)	51.24 ± 4.96 (6–75 ± 18.27)	
**Surgical procedure**			
Pneumonectomy	20 (24.7%)	50.69 ± 3.94 (4–75 ± 18.38)	0.151
Non-pneumonectomy	61 (75.3%)	61.99 ± 5.45 (5–75 ± 17.52)	

*Abbreviation*: *NS* Nutritional supplements

**Fig. 1:**
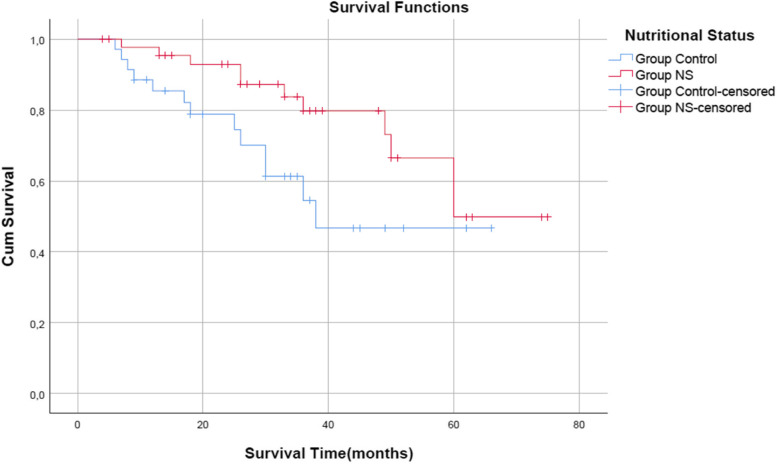
Survival analysis curve

The mean overall survival of the patients was 53.8 ± 3.3 (95% *CI*: 4.0–75.0) months, broken down into 58.9 ± 3.8 (95% *CI*: 4.0–75.0) months in Group_NS_ and 43.5 ± 4.6 (95% *CI*: 6.0–66.0) months in Group_C_. The mean survival was longer in Group_NS_ than in Group_C_, and the difference was statistically significant (*p* = 0.045) (Table 4) (Fig. 1).

The effects of parameters such as male gender, the presence of SCC, and nutritional status, whose effects on survival are known, on survival were examined by multivariate Cox regression analysis. This analysis revealed the risk of death was higher in men (*p* = 0.018, *HR*: 6.477 [1.375–30.503]) and those without nutritional support (*p* = 0.007, *HR*: 3.447 [1.407–8.449]) (Table 5).

**Table 5:** Evaluation of factors affecting survival by Cox regression analysis

**Patient groups**	**Hazard ratio**	**% 95 CL** **Lower–upper**	***p*-values**
Male gender	6.477	1.375–30.503	**0.018**
Squamous cell carcinoma	1.752	0.754–4.069	**0.192**
Nutritional support	3.447	1.407–8.449	**0.007**

## Discussion

Lung cancer is treated with a combination of surgical and oncological therapies but is still one of the leading causes of malignancy-related deaths. Due to the initially asymptomatic course and the metastatic nature of the disease, most cases are diagnosed in the advanced stages, thus removing the option of surgical treatment. In some of the cases that have a chance for surgical treatment, postoperative anticancer treatment methods (chemotherapy, radiotherapy, immunotherapy, or combinations) may be given according to the histopathological examination results of the surgical material and stage of the disease. Many studies have reported that anticancer treatments, especially chemotherapy treatment, have a positive effect on survival and reduce the risk of distant metastasis and local recurrence [3, 5–7, 11]. Despite these positive effects of chemotherapy, there are many adverse effects such as nausea, vomiting, diarrhea, other gastrointestinal system side effects, anorexia, malabsorption, weight loss, anemia, fatigue, electrolyte imbalance, cognitive disorders, neurotoxicity, nephrotoxicity, neutropenia, and increased risk of sepsis [12, 13]. In fact, some patients are lost due to these adverse effects.

Patients scheduled for postoperative chemotherapy must endure all the challenges of postoperative recovery as well as the adverse effects of chemotherapy, in addition to the presenting symptoms of the existing malignancy. The increased metabolic requirements following anatomic pulmonary resection become even more apparent under the catabolic effects of chemotherapy, and this process is also adversely affected by several factors, such as pain, anxiety, impaired taste, and loss of appetite, resulting in a considerable reduction in food intake [3, 10, 14]. This issue has also inspired various studies, and it has been shown that in patients with lung cancer, 30 to 73% of the patients have malnutrition and weight loss despite various treatment methods [15, 16]. In the PreMiO study conducted in 22 oncology centers in Italy, it was reported that 50% of cancer patients had malnutrition. It was found that while 40% of these patients were affected by anorexia, 60% were affected by weight loss [17]. When the appropriate nutritional support is not provided in this period, patients may experience biochemical changes, such as decreased levels of albumin-globulin in the blood. Losses of weight and muscle mass, delayed wound healing, and weakened immunomodulation, among the other problems experienced secondary to protein-energy malnutrition, can also be expected in later stages [3, 7–10, 14]. Recent studies show that malnutrition increases chemotherapy intolerance, morbidity, and mortality and decreases survival and quality of life [18–20]. For this reason, the provision of nutritional support to patients is a practical, effective, and cost-efficient approach to the effective management of this process [3, 14].

Despite the recent increase in the number of studies highlighting the importance of nutritional support in patients with malignancy, there have been only limited studies investigating the relationship between malignancy and lung surgery [8–10, 14, 21–25]. Additionally, most of the studies compare the preoperative nutritional status of patients within the postoperative period [21–25]. There have been studies reporting that patients receiving preoperative nutritional support develop fewer postoperative complications and are discharged earlier than those not receiving such support [14, 21–25]. Based on similar results, the European Society of Thoracic Surgeons (ESTS) has emphasized malnutrition as one of the leading risk factors behind the development of complications following lung surgery and recommend preoperative nutritional support. For the identification of patients eligible for nutritional support, the ESTS suggests following the ESPEN guidelines and recommends the initiation of preoperative enteral nutrition in high-risk patients that meet such criteria as “weight loss > 10–15% within six months, body mass index < 18.5 kg/m^2^ and serum albumin < 30 g/l,” as specified in the guidelines [2, 8]. In Park et al.’s [13] examination of 1011 cases with lung cancer using the “preoperative prognostic nutritional index” [10 × serum albumin (g/dl) + 5 × total lymphocyte count (/nl) based on the preoperative laboratory data], higher rates of postoperative complications and mortality were identified in cases with low prognostic nutritional index scores. Likewise, the study by Okada et al. [24], which included 505 cases operated due to NSCLC, identified an increased rate of postoperative pulmonary complications in cases with a low preoperative prognostic nutritional index. Lee et al. [25], in turn, used “the controlling nutritional status (CONUT)” tool in a series of 922 cases and reported similar results. The case series by Utsumi et al. [26] involving 108 patients undergoing pancreaticoduodenectomy found that patients with a high CONUT score were at greater risk of developing postoperative pancreatic fistula (a high CONUT score was associated with malnutrition). A review of literature reveals that different surgical units use different scoring systems for patient selection. In addition to nutritional status screening tests, there are also algorithms advocating the necessity of starting nutritional support based on clinical opinions in patients who are thought to have impaired food intake due to weight loss, body mass index, disease status, surgery, or anticancer treatment [27, 28].

The present study can be considered unique among earlier studies in literature. Despite the recognized importance of nutritional support in patients with malignancy, there is a lack of consensus on the optimum time period for the initiation of nutritional support [29]. In lung cancer, the question still remains of whether it should be initiated after diagnostic procedures, such as biopsies and transthoracic biopsies, or at a certain time prior to surgical treatment. In the present study, it was chosen not to administer nutritional support to patients who had been recently diagnosed with malignancy and prepared for surgical treatment, which seems reasonable, considering the fact that these patients were maintaining a normal life prior to surgery; were not exposed to any catabolic effect, such as chemotherapy-radiotherapy; had not faced any surgical stress; and had mostly only recently been diagnosed with malignancy. It was also considered that these patients would need to wait a little longer for wound healing before being exposed to the catabolic effects of postoperative chemotherapy. In this sense, it was concluded that the initiation of nutritional support early after the surgery would be both effective and more reasonable. It was further concluded that the similar rates of early postoperative complications between the two groups resulted from this approach. That said, it should be taken into account that many early postoperative complications are affected by other factors, such as the surgical treatment approach, the surgical technique, and comorbidities, and may not be directly associated with nutritional status. Nutritional therapy, it is worth noting, had a positive impact on survival, despite the later initiation when compared to other studies, which may indicate that nutritional support would have a positive effect in all periods in patients diagnosed with this malignancy.

The time of initiation of nutritional support, as well as the choice of nutritional support, is an important topic that needs to be explained. In recent years, instead of nutritional supports that meet the energy and protein needs of patients, high-energy, high-protein immunonutrition nutritional supports rich in arginine, glutamine, omega-3 fatty acids, and nucleotides that support the immune system are recommended. It has been reported that immunonutrition nutritional supports reduce the length of hospital stay and postoperative complications due to the anti-inflammatory effect of omega-3 fatty acids, and their inhibition of catabolic processes and treatment intolerance [30–32]. In addition, many studies have reported that immunonutrition nutritional supplements reduce intolerance and improve survival in anticancer treatments such as chemotherapy and immunotherapy [33–35]. Caccialanza et al. reported that those who received immunonutrition nutritional support had a higher tolerance to immunotherapy than the other group [36]. In our study, immunonutrition nutritional supplements were used, and no difference was observed between the groups in terms of complications and length of hospital stay. However, it was determined that survival was higher in patients who underwent immunonutrition nutrition. In addition, it was determined that the risk of death was 3.4 times higher in patients who did not receive nutritional support.

Another unique aspect of the present study is related to the selection of patients for the administration of nutritional support. Various approaches have been proposed in literature, and so there is still lack of standardization in this regard [21–25]. The present study also did not follow a standardized approach to patient selection, which leaves the method open to criticism. It could be seen, given the retrospective nature of the study, that the patients were selected based on the surgeon’s examination findings, comorbidities in the patient records, history of recent weight loss, biochemical test results, and, more importantly, the extent of the surgical treatment performed. Accordingly, it could be considered reasonable to provide nutritional support to the patients considered to be at the greatest need — in other words, those with a poor general status and those scheduled for more major surgeries. This can be clearly understood from the fact that 17 (85%) of the 20 pneumonectomy cases were in the group receiving nutritional support in the present study. In a similar vein, 21 (45.7%) of the 46 patients were at Stage III in Group_NS_ when compared to 37.1% in Group_C_, indicating the same approach. Despite this relative disadvantage to those in Group_NS_, it is a promising finding that the statistical parameters were similar between the two groups, and that survival was longer in Group_NS_.

## Conclusions

The body of knowledge on the treatment of lung cancer is increasing every day, but in spite of all the developments to date, the nature of the disease still lies beyond the abilities of a single discipline and demands a multidisciplinary approach. Despite being a relatively new concept in thoracic surgery, nutritional support has been observed to have favorable effects on patients undergoing surgery. We believe that the present study may serve as a guide in the development of an updated treatment approach.

### Limitations

There are some limitations of this study that should be kept in view when interpreting. Firstly, this is a retrospective and single-center study; therefore, the methodology used cannot be generalized to other centers. Secondly, the number of cases in the study was small but sufficient for statistical evaluation. To the best of our knowledge, this is the first and only study to highlight the significance of nutritional support in patients undergoing postoperative adjuvant chemotherapy for lung cancer. We thus believe that the present study makes a significant contribution to the literature and can lead other studies.

## Data Availability

The dataset of the research presented in this article is under record. Corresponding author can be contacted to reach the required data network.

## Abbreviations

C: Control
CONUT: The controlling nutritional status
COPD: Chronic obstructive pulmonary disease
CTx: Chemotherapy
ESTS: European Society of Thoracic Surgeons
NS: Nutritional supplements
NSCLC: Non-small cell lung cancer
SCLC: Small cell lung cancer
